# Prone positioning ventilation treatment rescuing a patient with chlamydia abortus-induced ARDS diagnosed by next generation sequencing: a case report

**DOI:** 10.3389/fmed.2024.1428300

**Published:** 2024-09-12

**Authors:** Yi Chen, Pin Lan, Lixue Liu, Kechun Zhou

**Affiliations:** ^1^Department of Emergency, Lishui Municipal Central Hospital, Zhejiang, China; ^2^Clinical Laboratory, BGI Genomics, Shanghai, China

**Keywords:** chlamydia abortus, prone positioning ventilation, pneumonia, acute respiratory distress syndrome, metagenomic next-generation sequencing

## Abstract

**Background:**

Chlamydia abortus is a pathogen capable of infecting both humans and animals. In most known cases, this pathogen primarily infects pregnant women, leading to miscarriage and preterm birth. However, it is exceedingly rare for this pathogen to cause pneumonia that progresses to severe Acute Respiratory Distress Syndrome (ARDS).

**Case introduction:**

We present a case of a 76-year-old male patient who was clinically diagnosed with Acute Respiratory Distress Syndrome (ARDS) caused by Chlamydia abortus and successfully treated. The patient’s condition rapidly deteriorated over six days, evolving from a lung infection to severe pneumonia, ultimately leading to ARDS and sepsis. Initially, he was admitted to a local hospital for a lung infection where routine etiological examinations failed to identify any significant pathogens, and he received only empirical antimicrobial therapy. However, the lung infection was not controlled, and the patient’s condition rapidly worsened, resulting in severe respiratory distress. This necessitated tracheal intubation and assisted ventilation, after which he was transferred to our hospital for treatment. Due to the patient’s family’s inability to afford the cost of ECMO treatment, we adopted a prone positioning ventilation strategy to improve the patient’s ventilation-perfusion matching. Additionally, we performed metagenomic next-generation sequencing on the patient’s bronchoalveolar lavage fluid, which confirmed the infection with Chlamydia abortus. These measures ultimately led to the successful treatment of the patient.

**Conclusion:**

Chlamydia abortus infection can lead to severe ARDS, necessitating timely diagnosis and active intervention by clinicians. This case highlights the crucial role of metagenomic next-generation sequencing in diagnosing rare pathogens. Timely adoption of prone positioning ventilation can significantly improve ventilation-perfusion matching, effectively treating ARDS caused by Chlamydia abortus. Additionally, the combination of moxifloxacin and piperacillin-tazobactam can treat ARDS caused by Chlamydia abortus.

## Introduction

Chlamydia abortus is a non-motile, obligate intracellular Gram-negative bacterium belonging to the Chlamydiaceae family. It primarily infects ruminants, particularly sheep and goats, and occasionally affects cattle, pigs, and horses. This pathogen can also infect humans, posing a particular threat to pregnant women, potentially leading to miscarriage, preterm birth, and sepsis (1–3). Although Chlamydia abortus mainly infects animals, it has gradually become a public health concern due to its potential risk to pregnant women and individuals with compromised immune systems (4). The clinical and radiographic presentations of pulmonary infections caused by Chlamydia abortus are similar to those caused by other pathogens, and routine laboratory tests (such as sputum culture, PCR) may not provide a definitive diagnosis, making it challenging to identify.

Globally, only six cases of pneumonia caused by Chlamydia abortus have been reported (3, 5–9). Among these cases, four patients developed severe ARDS, of whom only one, despite receiving extracorporeal membrane oxygenation (ECMO) treatment after unsuccessful prone positioning ventilation, unfortunately passed away (9). Prone positioning ventilation is a rescue therapy for patients with ARDS when conventional ventilatory support fails to maintain adequate blood oxygenation. Herein, we report a case of severe pneumonia caused by Chlamydia abortus infection. The patient developed ARDS and was treated with tracheal intubation and assisted ventilation, including prone positioning ventilation to improve ventilation-perfusion matching. The patient’s condition improved following treatment with antimicrobial agents such as imipenem-cilastatin sodium, moxifloxacin, and piperacillin-tazobactam.

## Case report

**FIGURE 1:**
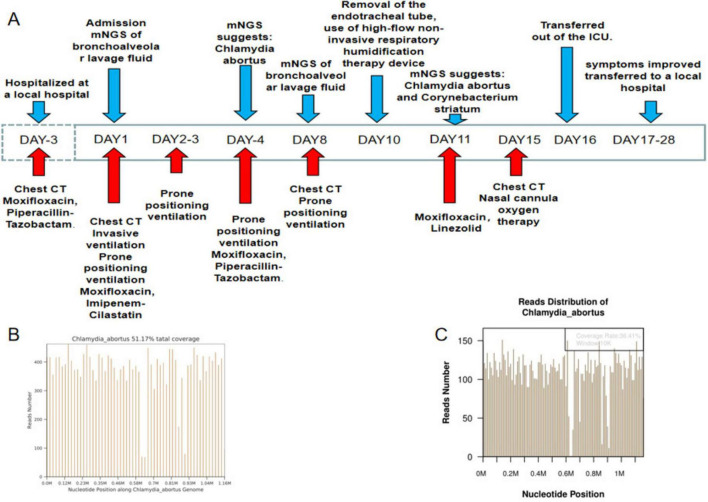
Patient’s treatment course and NGS sequencing results. **(A)** Timeline of the clinical condition progress and major management of the patient. **(B)** The patient’s first NGS results. **(C)** The patient’s second NGS results.

**FIGURE 2:**
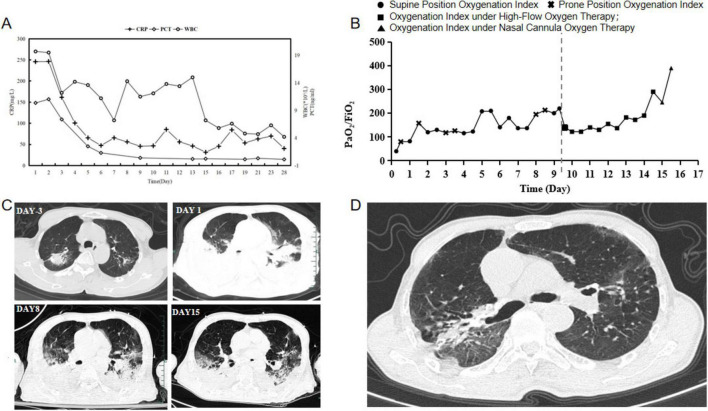
Patient’s blood tests and imaging results during treatment. **(A)** Trends in white blood cell count (WBC), procalcitonin (PCT), and C-reactive protein (CRP) during hospitalization. **(B)** Trends in oxygenation index during hospitalization. **(C)** Changes in chest imaging during hospitalization. **(D)** Chest imaging results one month after re-examination.

The patient was a 76-year-old healthy male who was admitted to the hospital due to persistent cough and fever for six days, which worsened on the day of admission. Six days before admission, he began to experience cough and high fever (39.5°C), along with chest tightness after activity, but did not seek immediate medical attention. Three days before admission, his symptoms worsened, and he visited a local hospital. Chest CT scans revealed scattered infectious lesions in both lungs. Despite receiving anti-infective treatments with moxifloxacin and piperacillin sodium tazobactam sodium, his symptoms did not improve, and routine etiological examinations did not yield any positive results. Treatment was then switched to imipenem-cilastatin sodium, but the patient’s condition did not improve and he experienced shortness of breath with a continuous decline in blood oxygen saturation. Consequently, he underwent tracheal intubation and was transferred to our hospital’s emergency department. Figure 1 is a timeline of the clinical condition progress and major management of the patient. The patient has no history of hypertension, diabetes, lung disease, AIDS, or other immunodeficiency diseases, no history of long-term medication use, no history of smoking or alcohol consumption, and engages in farming and raising chickens and ducks at home. Laboratory tests revealed arterial blood gas analysis (using 100% oxygen concentration) results: partial pressure of carbon dioxide 33.0mmHg, oxygen partial pressure 55.8mmHg, base excess −4.4 mmol/L, blood glucose 13.50 mmol/L, lactate 2.0 mmol/L; CRP 241.53 mg/L, white blood cell count 16.8 × 10^9/L, neutrophils 97.4%, lymphocytes 1.1%. Emergency coagulation function tests showed fibrinogen 8.40g/L, partial thromboplastin time 43.2 seconds, D-dimer 11.41mg/L, antithrombin III 67.3%. Chest X-ray indicated multifocal infections in both lungs, with large areas of consolidation, more pronounced in the right lung, and bilateral pleural effusion. The patient was admitted with a diagnosis of “severe pneumonia, acute respiratory failure.” Physical examination upon admission showed a temperature of 37.8°C, heart rate 98 beats per minute, respiratory rate 24 breaths per minute, blood pressure 131/98 mmHg, and weight 45kg, APACHE II score was 15 points. On the day of admission, the patient received intravenous infusion of imipenem-cilastatin sodium (1 g every 8 h) and moxifloxacin (0.4 g daily) for anti-infection treatment. Additionally, a bronchoalveolar lavage fluid sample was collected for metagenomic next-generation sequencing. Under the AC/PC mode of the ventilator (oxygen concentration 100%, respiratory rate 15 breaths per minute, tidal volume 400 mL, positive end-expiratory pressure 15.0 cmH_2_O), blood gas analysis showed a partial pressure of oxygen of 40 mmHg and an oxygenation index of 40. We recommended veno-venous extracorporeal membrane oxygenation (VV-ECMO) treatment for the patient, but his family declined due to financial reasons. Therefore, we opted for prone positioning ventilation. Two hours later, blood gas analysis showed a significant improvement in the patient’s oxygenation index to 80. On the fourth day of hospitalization, metagenomic next-generation sequencing results indicated a positive Chlamydia abortus infection (sequence number: 6286, relative abundance: 84.45%, gene coverage: 51.17%) (Figure 1B). Considering the significant improvement in CRP, procalcitonin, and oxygenation index, we discontinued imipenem-cilastatin sodium and switched to piperacillin-tazobactam (4.5 g every 8 h) combined with moxifloxacin (0.4g daily) for anti-infection treatment and temporarily stopped prone positioning ventilation. However, on the eighth day of hospitalization, the patient exhibited increased airway secretions and a decline in oxygenation index, prompting a repeat chest CT scan and intermittent prone positioning ventilation. At the same time, we recollected bronchoalveolar lavage fluid for metagenomic next-generation sequencing analysis. During treatment, the patient’s symptoms and indicators significantly improved. Finally, on the tenth day of hospitalization, the patient successfully underwent tracheal extubation and was switched to high-flow non-invasive respiratory humidification treatment. On the eleventh day, metagenomic next-generation sequencing results showed Chlamydia abortus infection (sequence number: 10951, relative abundance: 6.31%, gene coverage: 36.41%) (Figure 1C) combined with Corynebacterium striatum infection (sequence number: 455021, relative abundance: 74.98%), leading to a change in treatment to moxifloxacin (0.4g daily) combined with linezolid (0.6g every 12 h) for anti-infection treatment. After treatment, the patient’s inflammatory markers improved (Figure 2A), oxygenation index increased (Figure 2B), lung inflammation resolved (Figure 2C), and he was successfully transferred out of the ICU on the sixteenth day of hospitalization. The patient’s symptoms continuously improved during hospitalization, and after a period of treatment and rehabilitation, his condition stabilized, and he was transferred back to the local hospital for continued treatment. One month later, during a follow-up visit, his respiratory symptoms had almost disappeared, and chest imaging showed significant absorption and improvement (Figure 2D).

## Discussion

Chlamydia abortus is a zoonotic pathogen that primarily infects animals, but also readily infects pregnant women and individuals with compromised immune systems. Human infections with Chlamydia abortus were extremely rare until Ortega et al. reported a case of pneumonia caused by this pathogen (5). The case presented here is a rare instance of ARDS triggered by Chlamydia abortus. Due to the patient’s inability to afford the costs of veno-venous extracorporeal membrane oxygenation (VV-ECMO), we managed to significantly improve his condition through active anti-infective treatment and prone positioning ventilation. Early use of prone positioning ventilation has been shown to increase survival rates in patients with moderate to severe ARDS (10). In patients with ARDS, prone positioning helps re-expand collapsed lung tissue in the dorsal regions while reducing over-ventilation in ventral areas, thus facilitating repositioning of lung tissues and achieving more uniform lung ventilation. This more even distribution of ventilation helps reduce the risk of ventilator-induced lung injury (11). Although there are no reports on improving the oxygenation index of ARDS caused by Chlamydia abortus through prone position ventilation, studies have shown that prone position ventilation can significantly improve the oxygenation index in most patients with COVID-19-induced ARDS (12). Given the potential of this therapy to improve the oxygenation index, we employed a series of combined treatment approaches in this case, including ventilator support, prone position ventilation, and targeted antibiotic use, which ultimately led to significant improvement in the patient’s condition.

Currently, the primary diagnostic method for Chlamydia abortus infection is Polymerase Chain Reaction (PCR) (4). This technique, highly specific, is usually performed only when there is a high clinical suspicion of Chlamydia abortus infection. In this study, traditional detection methods failed to identify any pathogens, posing a significant challenge for accurate diagnosis and precise treatment. The advantage of metagenomic next-generation sequencing is that it sequences all nucleic acids in a sample, characterized by speed, high accuracy, and wide coverage. Research reports have shown high sensitivity in the diagnosis of psittacosis and Chlamydia abortus using metagenomic next-generation sequencing (13). In this case, we collected bronchoalveolar lavage fluid samples from the patient and performed metagenomic next-generation sequencing. The results detected Chlamydia abortus, providing guidance for our subsequent treatment strategies. Reviewing the case, it is highly likely that the patient contracted the pathogen while raising poultry.

Globally, there have been only four reported cases of ARDS caused by Chlamydia abortus infection. Due to the relative rarity of such cases, there are no specific guidelines for the treatment of Chlamydia abortus infection. Case reports suggest that Chlamydia abortus infections often involve mixed infections, hence the recommendation for combined treatment with moxifloxacin and piperacillin-tazobactam (6). In this case, although the patient initially received piperacillin-tazobactam, imipenem-cilastatin, and moxifloxacin treatment at the local hospital, his lung function continued to deteriorate, possibly due to the progression of the disease or insufficient treatment duration. After we identified the pathogen through metagenomic next-generation sequencing, we also used a combination of moxifloxacin and piperacillin-tazobactam. Due to the severity of the patient’s infection, we administered piperacillin-tazobactam (4.5g every 8 h) combined with moxifloxacin (0.4g daily) for a total of 8 days, followed by moxifloxacin alone (0.4g daily) for 5 days. Eventually, the patient’s pulmonary infection lesions significantly regressed, the oxygenation index improved, invasive mechanical ventilation was successfully discontinued, and the patient ultimately recovered and was discharged. Given the rarity of pulmonary infection cases caused by Chlamydia abortus and the considerable variability in disease severity and individual patient differences (3, 5–9), the duration of antibiotic use should be tailored to the specific condition. This case serves as a reference only.

## Conclusion

The early application of prone positioning ventilation, combined with the anti-infection treatment regimen of moxifloxacin and piperacillin-tazobactam, can effectively treat ARDS caused by Chlamydia abortus. This also underscores the significant role of metagenomic next-generation sequencing in the diagnosis of rare pathogens.

## Data Availability

The original contributions presented in the study are included in the article/supplementary material, further inquiries can be directed to the corresponding author.
